# Identification of the Combinatorial Effect of miRNA Family Regulatory Network in Different Growth Patterns of GC

**DOI:** 10.1016/j.omto.2020.03.012

**Published:** 2020-03-30

**Authors:** Jia Cheng, Huiqin Zhuo, Lin Wang, Wei Zheng, Xin Chen, Jingjing Hou, Jiabao Zhao, Jianchun Cai

**Affiliations:** 1 Department of Gastrointestinal Surgery, Zhongshan Hospital, Xiamen University, Xiamen, Fujian 361004, China; 2 Institute of Gastrointestinal Oncology, School of Medicine, Xiamen University, Xiamen, Fujian 361004, China; 3 Xiamen Municipal Key Laboratory of Gastrointestinal Oncology, Xiamen, Fujian 361004, China; 4 Union Hospital, Fujian Medical University, Fuzhou, Fujian 350001, China

**Keywords:** gastric cancer, COL4A1, Ming’s classification, biomarker, miR-29s, ECM-receptor interaction, survival analysis, histological classification, TCGA database, gene regulatory networks

## Abstract

According to the growth pattern, gastric cancer (GC) could be classified into expanding-type GC and infiltrative-type GC (Ming’s classification). The growth pattern of GC is often related to the malignant degree, invasion, metastasis, and other pathological characteristics of tumors. MicroRNAs (miRNAs) play important roles in modulating gene expression during the GC development. In this study, miR-29s were significantly correlated with the gastric carcinogenesis and Ming’s classification. Biological function of miR-29s is most closely related to the pathway of extracellular matrix (ECM)-receptor interaction. ECM structural assembly, cell movement, and cell adhesion are the main functional categories of target genes in this pathway. Among these targets, the COL4A1 gene ranked at the top in the association analysis of combined miR-29s biological function and GC subtype, and miR-29s inhibited its translation by binding to the 3′ UTR region. Infiltrative-type GC cells secrete a higher level of COL4A1 protein than do expanding-type GC cells. The expression of COL4A1 in GC is correlated with clinicopathological features. Downregulation of COL4A1 expression significantly inhibited the migration and invasion of GC cells. High COL4A1 expression was correlated with poor prognosis in survival analysis. The miR-29s regulatory network may affect the development of growth patterns and pathological progress of GC by regulating the function of COL4A1.

## Graphical Abstract

**Figure:**
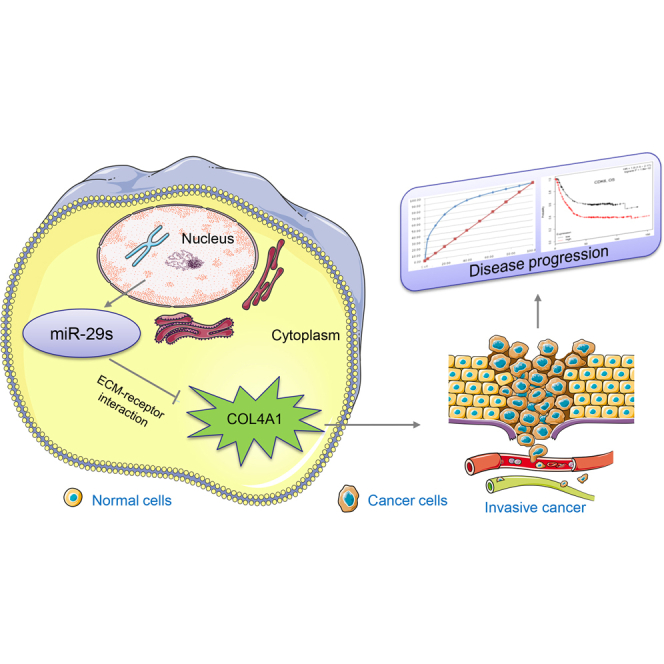

## Teaser

According to the growth pattern, gastric cancer (GC) could be classified into expanding-type GC and infiltrative-type GC. Cai and colleagues show that miR-29s were differentially expressed in these two types of GC and mainly regulated the ECM-receptor interaction pathway by targeting COL4A1 to inhibit the migration and invasion of GC cells.

## Introduction

Gastric cancer (GC) is an important public health problem throughout the world.^1^ There are about 1 million new cases of GC in the world every year, especially in Asia, where the incidence of GC is high.^2^ The difficulty of early diagnosis and the lack of biological markers have been the key factors restricting treatment of GC. At present, due to various reasons, most countries have not carried out early screening of GC, and there are no specific symptoms in the early stage of GC, which makes GC often progress to an advanced stage before diagnosis. The overall 5-year survival rate of early stage patients with GC is about 90% after reasonable treatment, while the rate of advanced GC patients is greatly reduced.^3^^,^^4^ Based on the different patterns of cell growth and infiltration, GC could be accurately classified into expanding-type GC and infiltrative-type GC, i.e., Ming’s classification.^5^ Expanding-type GC presents a mass-like expansive growth pattern, often forming numerous discrete tumor nodules in the tumor tissue, while infiltrative-type GC cells show a dispersed infiltration growth, without obvious aggregation.^5^ Ming’s classification is not only based on the structural pattern of tumor tissue, but it also combines the biological characteristics of cancer cell growth. This GC classification is not limited by the location and size of tumor growth, which can provide a complete picture of the biological development of GC as the basis of clinical prognosis evaluation combined with tumor-node-metastasis (TNM) staging system.^5^^,^^6^

Although some risk gene mutations have been identified, little is known about the exact molecular biological mechanism and the composition of risk gene regulatory networks that lead to GC occurrence. MicroRNA (miRNA) is an endogenous small RNA molecule with a length of about 18–25 nt.^7^^,^^8^ They can regulate the function of messenger RNA (mRNA) mainly by forming RNA-induced silencing complex (RISC) with other proteins such as Dicer.^9^ The 3′ untranslated regions (3′ UTRs) of mRNA are the most common binding site of miRNAs.^9^ In humans, about 30% of genes are regulated by miRNAs.^10^ The imbalance of the expression of miRNAs can act as oncogenes or tumor suppressor genes and participate in a series of lethal pathological mechanisms in GC, including tumor growth, microangiogenesis, cancer stem cell dimension, cancer cell metastasis, and chemotherapy resistance.

There are significantly differential gene regulation patterns between the expanding-type GC and infiltrative-type GC, which provide a good histological model for studying the biological principles of growth and invasion of GC.^11^ From this pathological view, it is helpful to find cancer-promoting or anti-cancer factors that play different biological roles in GC development. For example, the expression level of miR-145 in infiltrative-type GC is significantly lower than that in expanding-type GC, and miR-145 can inhibit the migration and invasion of GC cells by targeting FSCN1 (fascin actin-bundling protein 1).^12^ The levels of miR-15a-3p and miR-16-1-3p in infiltrative-type GC were significantly lower than those in expanding-type GC.^13^ Their common target gene TWIST1 (twist-related protein 1) was higher in infiltrative-type GC than that in expanding-type GC.^13^ These findings indicated that the regulatory network of miRNAs is not only involved in the occurrence of GC, but it also can regulate the growth pattern of GC, which leads to obvious individual differences in histopathology and morphology that greatly increases the difficulty of GC treatment. Some miRNAs have been found to be associated with specific histological subtypes of tumors.^14^^,^^15^ However, there is rare information about the differential of molecular regulatory mechanisms in the histological classification of GC that need to be further explored.

At present, many reports have confirmed that miR-29s are an important regulatory factor in GC development and are involved in the proliferation, metastasis, and drug resistance of GC cells.^16^^,^^17^ However, there is still a lack of reliable identification methods and sufficient research evidence about the most important biological role of its regulatory network in GC. We previously found that there are significant differential expression levels of this miRNA family between Ming’s classifications in GC.^11^ In previous miRNA microarray analysis, the expression of miR-29b/c is specifically low in infiltrative-type GC,^11^ but not significantly different in expanding-type GC.^11^ They belong to the same miRNA family with similar sequences and biological functions.^17^ This study is based on the important biological characteristics of different growth patterns of GC. A series of research methods were used to further clarify the pathological role of miR-29s in GC development and explore the important miR-29s regulatory pathways in two different types of GC according to Ming’s classification. The pathway function was accurately classified by various bioinformatics analysis methods, and then the biological mechanism of targeted genes was verified in GC cell lines from Ming’s classification, so as to clarify their effects on the biological behavior of GC cells. Finally, we evaluated the potential clinical significance of these GC growth pattern-related interacting molecules in the assessment of pathological progression and prognosis of GC. This study is helpful to a further understanding of GC development and exploring better diagnosis and treatment strategies for GC.

## Results

### Expression of miR-29s Was Downregulated in GC Tissues and Most Closely Related to the Pathway of Extracellular Matrix (ECM)-Receptor Interaction in Biological Function

**Figure 1:**
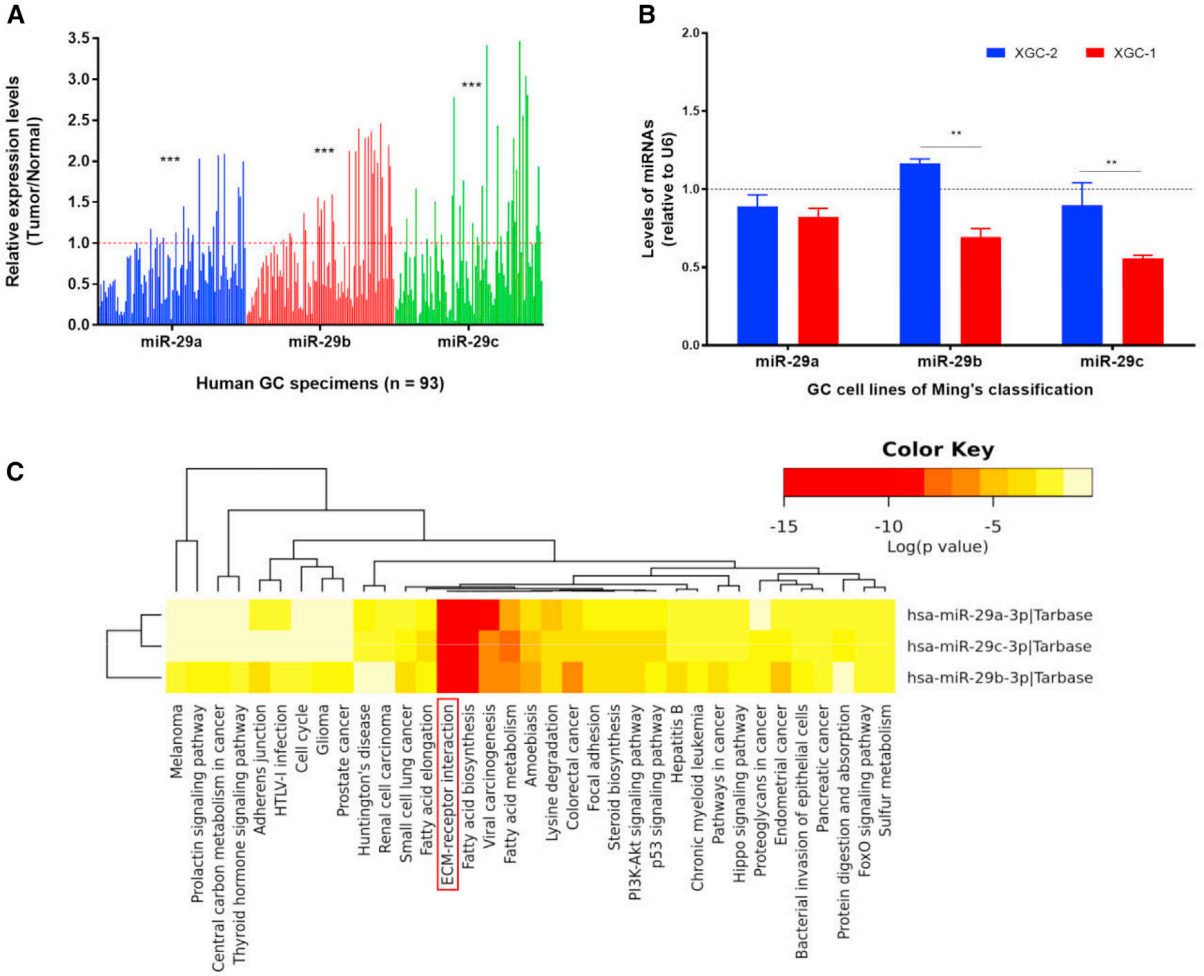
Expression Levels of miR-29s and Their Pathway Prediction (A) Expression levels of miR-29s in GC specimens. (B) miR-29s expression in cell lines. (C) Statistically significant correlations were revealed between miR-29s and their mediated pathways by p value (log scaled) in a heatmap. Red represents high significance.

This study focused on the role of all three members of the miR-29 family (miR-29s) in different growth patterns of GC. First, in order to further clarify the expression of miR-29s in GC, we further validated the previous results of miRNA microarray by enlarging the sample size of GC. The results showed that the miR-29s expression levels in GC tissues were significantly lower than those in paired normal gastric epithelium tissues (Figure 1A, ∗∗∗p < 0.001). In cell lines, miR-29b/c showed a differential expression between XGC-1 (infiltrative type) and XGC-2 (expanding type) (Figure 1B, ∗∗p < 0.01). By analyzing a series of bioinformatics databases, it was found that miR-29s were involved in many biological pathways (Figure 1C). The ECM-receptor interaction ranked the most significant pathway, and many target genes in this pathway were regulated by miR-29s (Table 1). Because of this, many members of the ECM family could influence the biological behavior of cancer cells. Therefore, we speculate that the ECM-receptor interaction pathway may be the main pathway for the biological function of miR-29s. This provides a direction for the subsequent screening of related target genes of miR-29s.

**Table 1:** Statistical Significance in Pathways Mediated by miR-29s

KEGG Pathways	Count	p Value
ECM-receptor interaction	25	<1e−325
Fatty acid biosynthesis	2	<1e−325
Viral carcinogenesis	45	<1e−325
Fatty acid metabolism	6	<1e−325
Amoebiasis	23	2.84E−11
Colorectal cancer	23	5.37E−11
Lysine degradation	10	5.59E−10
Focal adhesion	49	8.93E−09
Steroid biosynthesis	3	2.88E−08
PI3K-Akt signaling pathway	66	7.95E−08
p53 signaling pathway	17	1.88E−07
Endometrial cancer	16	9.08E−07
Small-cell lung cancer	25	9.12E−07
Fatty acid elongation	3	2.21E−06
Hepatitis B	33	4.66E−06
Pathways in cancer	74	6.47E−06
Hippo signaling pathway	27	7.96E-05
Chronic myeloid leukemia	19	8.23E−05
Bacterial invasion of epithelial cells	18	0.0003135
Pancreatic cancer	17	0.000584
Sulfur metabolism	2	0.0016031
FoxO signaling pathway	28	0.0016299

PI3K, phosphatidylinositol 3-kinase.

### Functional Enrichment Reveals That ECM Structural Assembly, Cell Movement, and Cell Adhesion Are the Main Functional Categories of Target Genes in This Pathway

**Figure 2:**
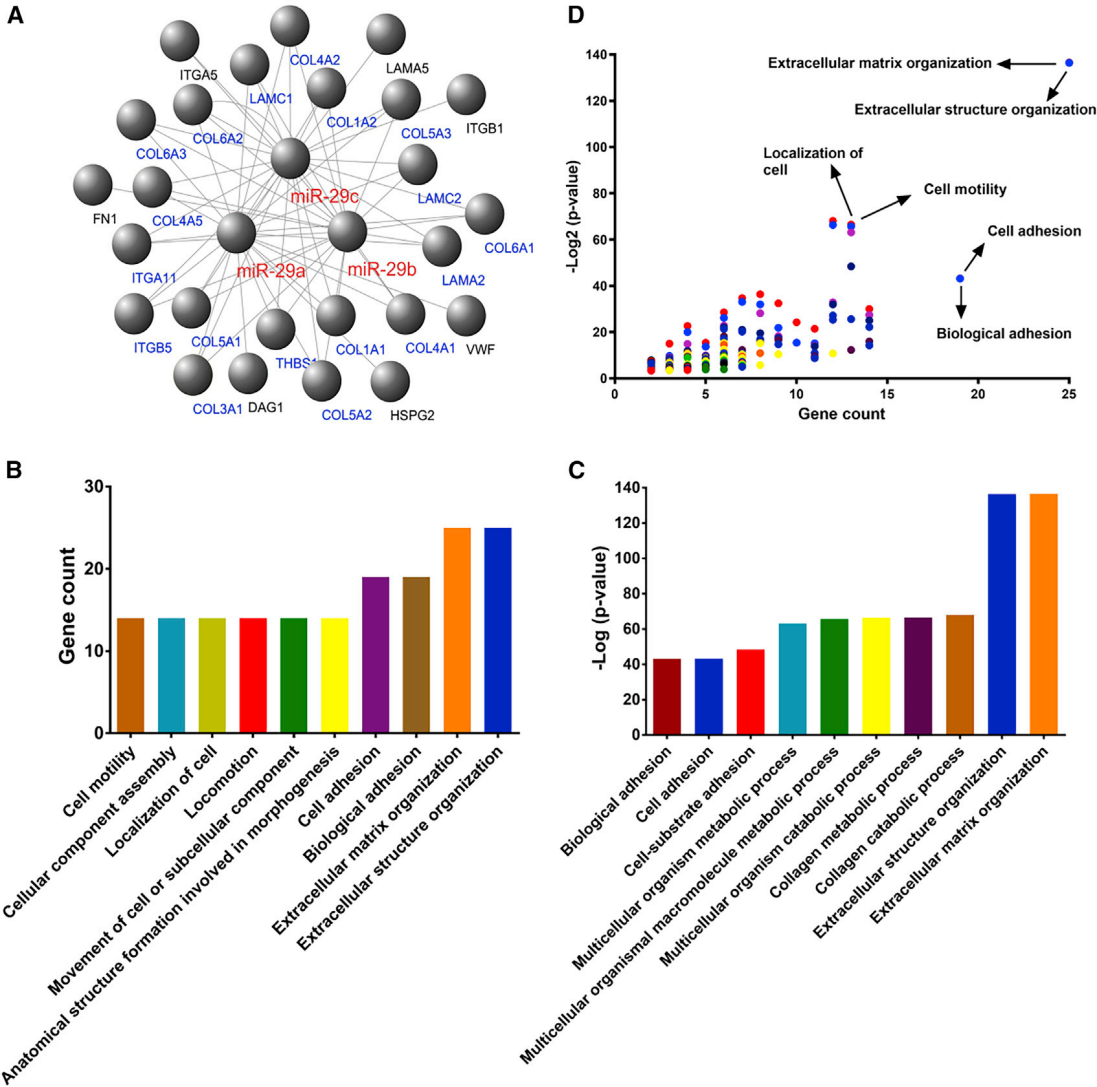
Function Annotations for Target Genes Mediated by miR-29s in KEGG Pathway Analysis (A) Establishment of a miR-29s-mRNA interactions network of ECM-receptor interaction. (B) The gene number of each cluster in the DAVID function annotation for the miR-29s-targeted genes of the ECM-receptor interaction. (C) The p value transformed by −log_2_ in the above-mentioned DAVID function annotation. The horizontal axes shows the annotated functions of the target genes. Only the most significantly enriched clusters are shown. (D) All cluster features about p value and gene count were demonstrated by the scatterplots, and the top right plots represent high significance and more genes. Most of them are concentrated in ECM organization, cell motility, and cell adhesion.

The relationship between the ECM-receptor interaction pathway and regulation of miR-29s was analyzed by DIANA-miRPath data mining. A total of 25 genes were regulated by miR-29s in this pathway. Cytoscape, a graphical interaction molecule display software, was used to construct a network of interactions between miR-29s and target genes in the ECM-receptor interaction pathway (Figure 2A). As shown, some genes are regulated by more than two molecules of miR-29s (Figure 2A). The target genes of miR-29s in this pathway were enriched and analyzed by DAVID tools to explore their main biological functions. Cluster analysis of many functional pathways on the basis of gene function enrichment is conducted to further identify the cellular biological mechanism of these target genes in the tumor microenvironment. We found that their functions mainly consist of three functional clusters: cell adhesion, endothelial development, and microangiogenesis (Table 2). In addition, the top 10 functional clusters with the gene counts involved in the interaction network and the statistical significance were graphically displayed to show the various biological functions. The results showed that ECM organization, cell motility, and cell adhesion were the main biological functions of the target genes of miR-29s, which was basically consistent with the results of the above-mentioned functional annotations of gene clustering (Figures 2B–2D).

**Table 2:** Functional Annotation Clustering of miR-29s Targets in the ECM-Receptor Interaction Pathway

Annotation Category	Count	%	−Log_2_ (p Value)
**Cluster 1**			

Cell-substrate adhesion	13	52	48.44
Cell adhesion	19	76	43.22
Biological adhesion	19	76	43.13

**Cluster 2**			

Endoderm development	8	32	36.39
Endodermal cell differentiation	7	28	34.70
Endoderm formation	7	28	33.05
Formation of primary germ layer	8	32	31.99
Anatomical structure formation involved in morphogenesis	14	56	29.99
Gastrulation	8	32	28.17
Embryonic morphogenesis	10	40	24.23
Embryo development	11	44	21.48
Regulation of anatomical structure morphogenesis	8	32	10.90

**Cluster 3**			

Blood vessel development	12	48	32.86
Vasculature development	12	48	31.98
Anatomical structure formation involved in morphogenesis	14	56	29.99
Cardiovascular system development	12	48	25.35
Circulatory system development	12	48	25.35
Blood vessel morphogenesis	9	36	21.86
Angiogenesis	8	32	19.53
Cellular response to growth factor stimulus	8	32	15.83
Response to growth factor	8	32	15.47
Leukocyte migration	6	24	12.52

### COL4A1 Gene May Play an Important Role in Biological Function of miR-29s

**Figure 3:**
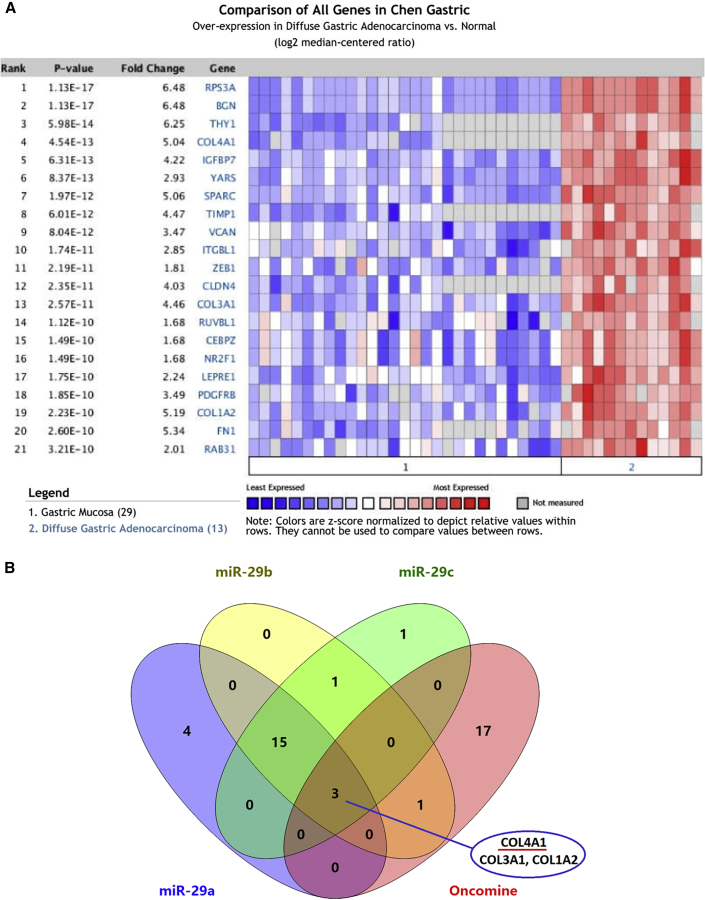
Screening of miR-29s Targets in Diffuse GC (A) High-Expression risk genes in diffuse GC of Oncomine database. (B) Venn Screening between above-mentioned High-Expression Risk Genes and miR-29s Target Genes in the ECM-Receptor Interaction Pathway

Owing to the lack of specific disease information about the functional enrichment of miR-29s target genes, it is necessary to explore the relationship between miR-29s target genes and gastric carcinogenesis in the ECM-receptor interaction pathway to reveal the role of these genes in the pathophysiological mechanism of GC development. Previous studies have proved that the expression of miR-29b/c is significantly low in GC, especially in infiltrative-type GC. Therefore, we compared and analyzed the target genes of miR-29s in the ECM-receptor interaction pathway by using the Oncomine database to explore their relationship with specific histopathological subtypes of GC. In Ming’s classification, diffuse GC can be classified as infiltrative-type GC. Therefore, this study excavated the microarray expression data with the largest sample size in a single study of diffuse GC for subsequent analysis. The results showed that three of miR-29s target genes in the ECM-receptor interaction pathway belonged to high-risk genes with higher expression in diffuse GC (Figure 3). Among them, the gene encoding human collagen type IV α1 chain (COL4A1) showed the highest expression (Figure 3A). Combining the above results, it is suggested that the target gene of miR-29s in the ECM-receptor interaction pathway may be involved in the formation of diffuse GC by affecting the biological functions of GC cells, such as cell movement and cell adhesion. This also indicated that the downregulated miR-29s may promote the histological origin of diffuse GC in view of their target gene function, suggesting that the target gene, especially COL4A1, may play an important biological role in this histopathological process.

### miR-29s Inhibited COL4A1 Translation by Binding to the 3′ UTR Region

**Figure 4:**
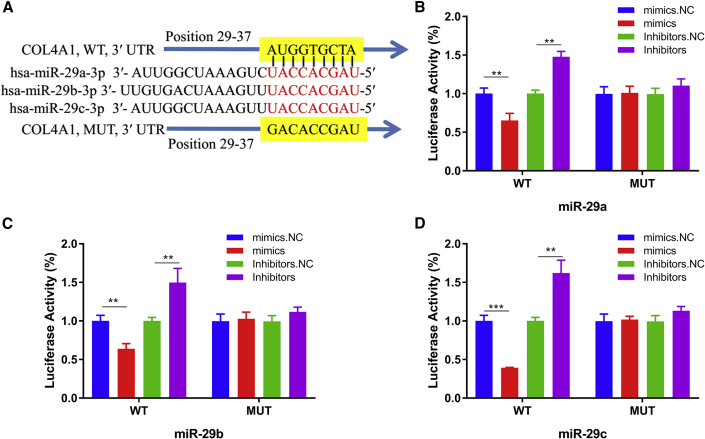
Identification of COL4A1 as a Direct Target of miR-29s (A) Yellow represent binding sites of the 3′ UTR of COL4A1 targeted by miR-29s. (B–D) HEK293T cells were co-transfected with LUC reporter plasmids containing either WT or MUT COL4A1 3′ UTR with miR-29s mimics, inhibitors, and the NCs. The relative LUC levels were detected 36 h after transfection. (B) miR-29a, (C) miR-29b, and (D) miR-29c.

**Figure 5:**
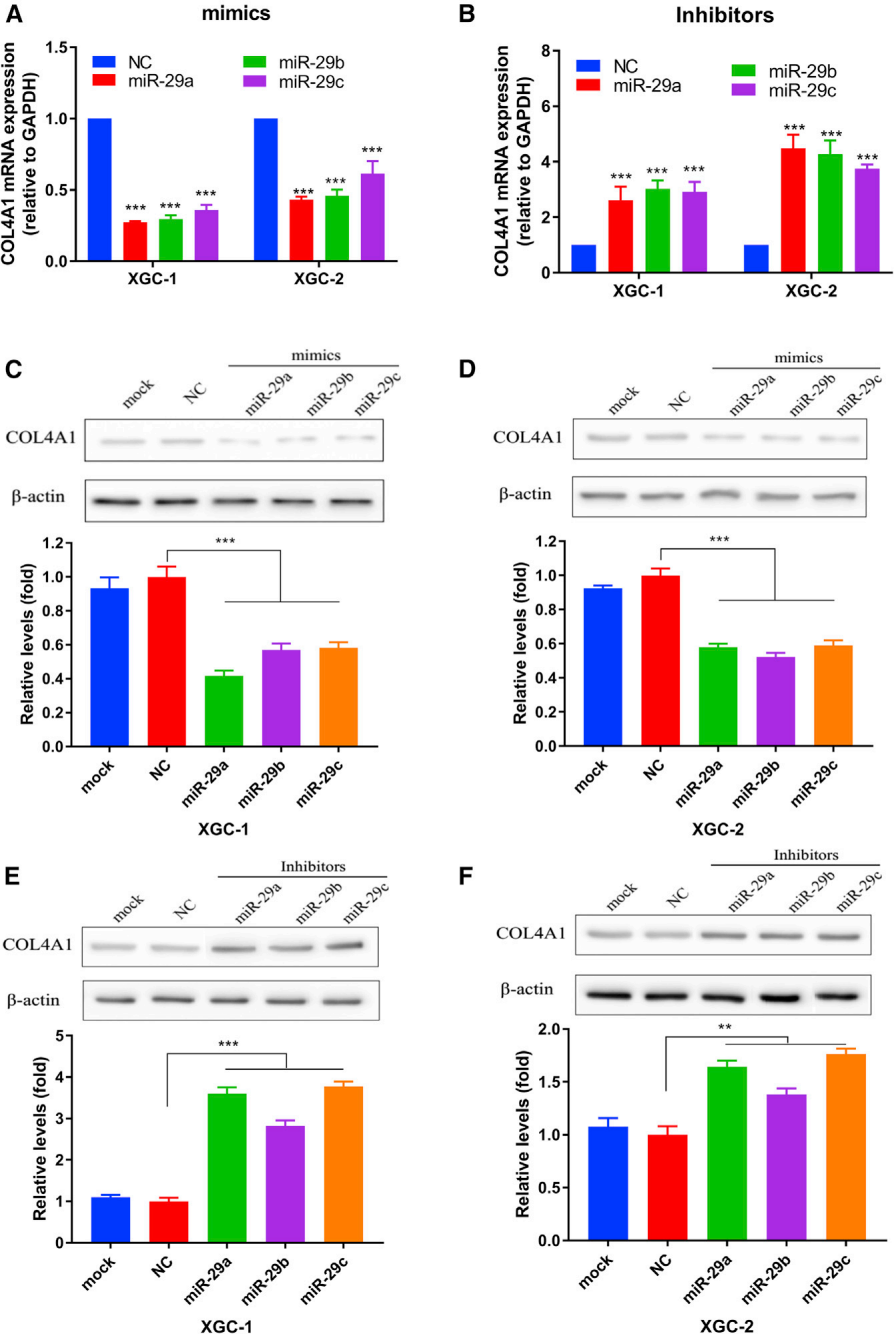
Analysis the Association between COL4A1 Expression and the Levels of miR-29s in GC Cells of Ming’s Classification. (A) miR-29s mimics reduced the mRNA level of COL4A1. (B) Inhibitors of miR-29s increased the mRNA level of COL4A1. (C) miR-29s mimics reduced the COL4A1 protein level in the XGC-1. (D) miR-29s mimics reduced the protein level of COL4A1 in the XGC-2. (E) Inhibitors of miR-29s increased the protein level of COL4A1 in the XGC-1. (F) Inhibitors of miR-29s increased the COL4A1 protein level in the XGC-2.

The experimental results showed that miR-29s mimics could significantly reduce the luciferase (LUC) activity of COL4A1 recombinant plasmid in the wild-type (WT) group, while the LUC activity in the WT group increased significantly after adding miR-29s inhibitors (Figure 4). This may be due to the decrease of endogenous miR-29s activity in cells caused by the miR-29s inhibitors, which increased the expression of LUC in WT recombinant plasmids. In the group of the COL4A1 3′ UTR mutant (MUT), either the addition of miR-29s mimics or inhibitors could not significantly impact the activity of LUC (Figure 4). This indicated that the MUT site reduced the ability of miR-29s to binding the COL4A1 3′ UTR region, and it suggested the molecular biological mechanism of miR-29s induced gene silencing of COL4A1. By detecting the changes of COL4A1 gene expression and protein level in GC cells transfected with inhibitors or mimics of miR-29s, the relationship between miR-29s and the COL4A1 gene was evaluated at the levels of both post-transcription and translation. The results showed that miR-29s mimics significantly reduced the COL4A1 mRNA level (Figure 5A). Correspondingly, the expression of COL4A1 increased significantly after transfection of miR-29s inhibitors in GC cells (Figure 5B). Western blotting (WB) showed that the COL4A1 protein level was significantly decreased in GC cells after being transfected with miR-29s mimics, while the COL4A1 protein level was significantly increased in GC cells transfected with transfection inhibitors (Figures 5C–5F). These results suggested that the COL4A1 gene may be a direct target of miR-29s, suggesting that miR-29s may participate in the histopathological process of GC different growth patterns by inhibiting the target gene expression in the ECM-receptor interaction pathway, especially the COL4A1 gene.

### Upregulated COL4A1 Levels Were Correlated with miR-29s in GC Tissues

**Figure 6:**
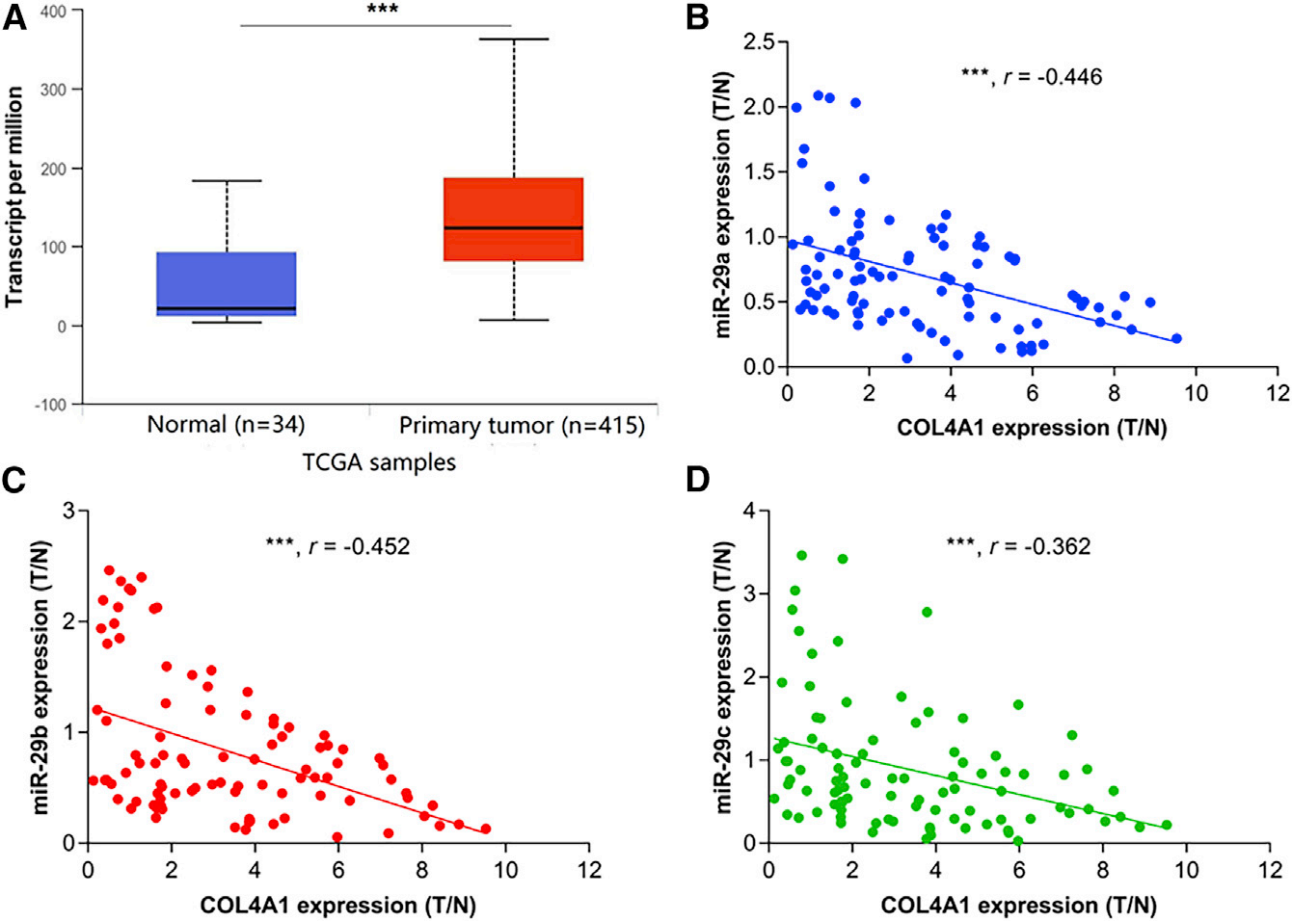
Upregulated COL4A1 Level Is Correlated with Low Levels of miR-29s in GC (A) Analysis of COL4A1 expression in GC using TCGA data. (B–D) The relationship between the COL4A1 expression and the levels of the three members of miR-29s in the enrolled samples, respectively. (B) miR-29a, (C) miR-29b, and (D) miR-29c. Tissues ∗∗∗p < 0.001.

We used the data mining tool UALCAN (http://ualcan.path.uab.edu/index.html) to analyze and verify the expression statues of the COL4A1 gene in GC tissues and normal gastric tissues with The Cancer Genome Atlas (TCGA) database. The results showed that the COL4A1 expression in GC tissues was significantly higher than that in normal gastric tissues (Figure 6A). In order to explore whether miR-29s are involved in regulating COL4A1 expression in clinical GC tissues, we investigated the relationship between miR-29s and the COL4A1 expression level in 93 paired GC tissues and normal gastric epithelium tissues collected from Zhongshan Hospital. There was a significant correlation between miR-29s and COL4A1 expression levels (Figures 6B–6D). These results suggested that all three members of miR-29s participated in the regulation of COL4A1 gene expression in GC tissues. The miRNA-mRNA paired interaction molecule may be involved in the clinical development of GC and regulation of different growth patterns of GC.

### Meta-Analysis Confirmed That the High Expression of the COL4A1 Gene Promotes the Development of GC

**Figure 7:**
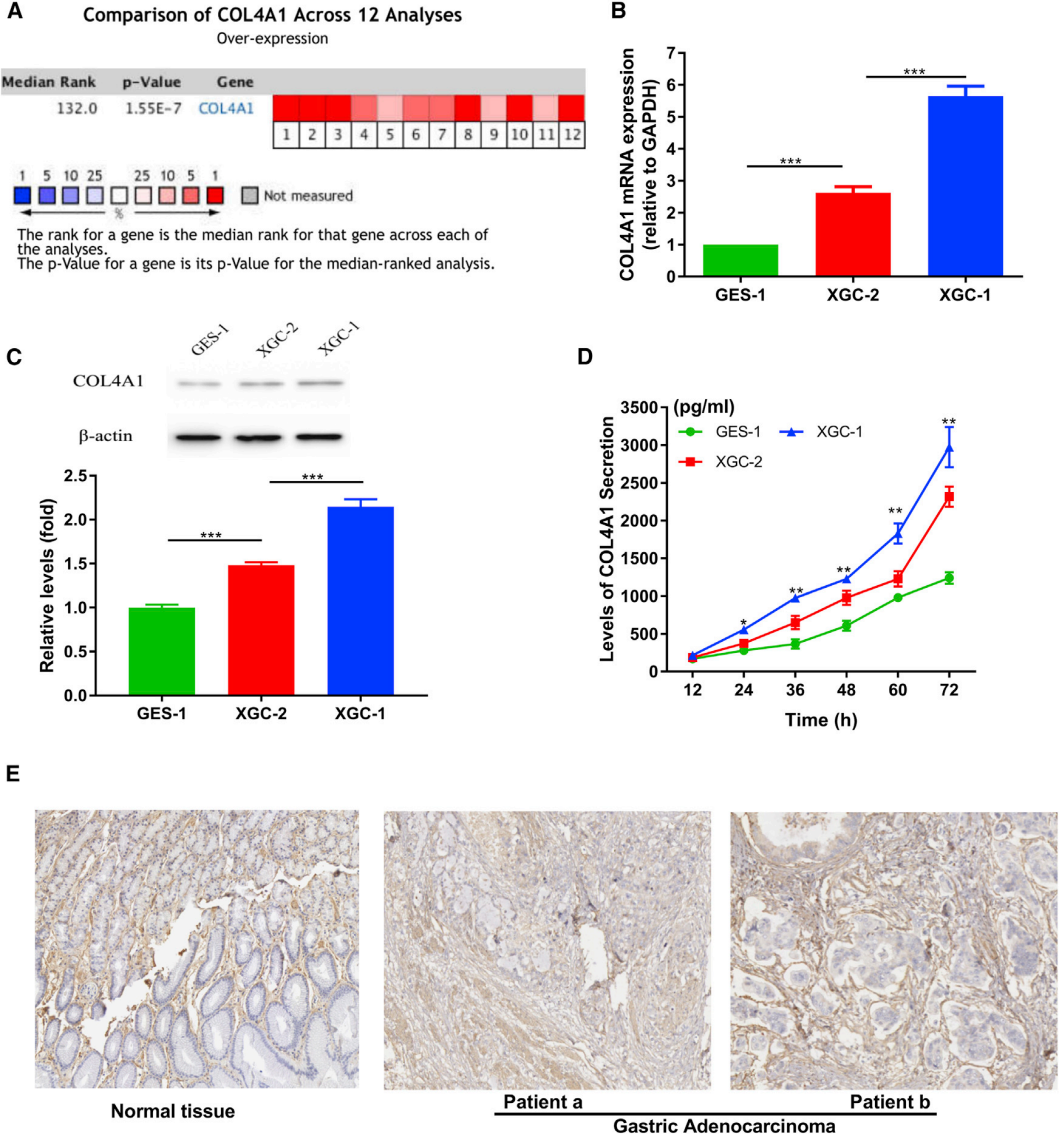
The Expression of COL4A1 Was Significantly Different According to GC Cells of Ming’s Classification (A) Meta-analysis of COL4A1 gene expression in GC. (B) The mRNA levels of COL4A1 in the infiltrative GC cells was significantly higher than that in the expanding GC cells and normal gastric epithelial cells. (C) The COL4A1 protein expression in the infiltrative GC cells was also significantly higher than that in the expanding GC cells and normal gastric epithelial cells. (D) ELISA method was used to detect the different extracellular secretion levels of COL4A1 protein in GC cells of different growth patterns. (E) Immunohistochemical database analysis showed a large accumulation of COL4A1 protein in gastric adenocarcinoma tissues. ∗∗∗p < 0.001.

Gene expression detection in a single study is vulnerable to the influence of technical methods, genetic background, and sample size, which may lead to errors in conclusions. In order to eliminate this kind of influence on the identification of COL4A1 gene expression in GC, we conducted a meta-analysis of COL4A1 gene expression in different technology platforms and genetic backgrounds by using several microarray databases of GC. A total of 12 results (n = 298) from from datasets of Oncomine were included in the meta-analysis. The result showed that COL4A1 gene was generally highly expressed in GC (Figure 7A). Thus, it was clear that the COL4A1 gene regulated by miR-29s is not only involved in the carcinogenesis of GC, but it also may affect the occurrence of different growth patterns of GC.

### Infiltrative-type GC Cells Secrete Higher COL4A1 Protein Levels Than that in Expanding-type GC Cells

In this study, the expression of COL4A1 in multiple GC cell lines was detected (Figures S1A and S1B). Expression of COL4A1 in two GC cell lines from different Ming’s classification was significantly higher than that in gastric epithelial cells (Figures 7B and 7C), and the expression of COL4A1 in infiltrative-type cell lines (XGC-1) was significantly higher than that in expanding-type GC cell lines (XGC-2). As an important component of ECM structure in histogenesis, COL4A1 protein exists both inside and outside cells. The protein level of COL4A1 in cell culture medium was detected by ELISA technology, so as to evaluate the ability to secrete COL4A1 protein in different Ming’s classification GC cells and provide a preliminary basis for further exploring the effect of COL4A1 protein on biological behavior of GC cells. ELISA showed that the GC cells secreted a higher level of COL4A1 protein than that in gastric epithelial cells, and the XGC-1 cells secreted a higher level of COL4A1 protein than that in XGC-2 cells (Figure 7D). The COL4A1 protein aggregation in GC interstitial space was higher than that in normal gastric tissue, which was shown in a human protein immunohistochemical database of gastric epithelial tissue and GC tissue (Figure 7E).

### COL4A1 Expression Is Correlated with Clinicopathological Features Such as Tumor Subtype, Malignant Degree, Disease Stage, and *Helicobacter pylori* (HP) Infection in GC

**Figure 8:**
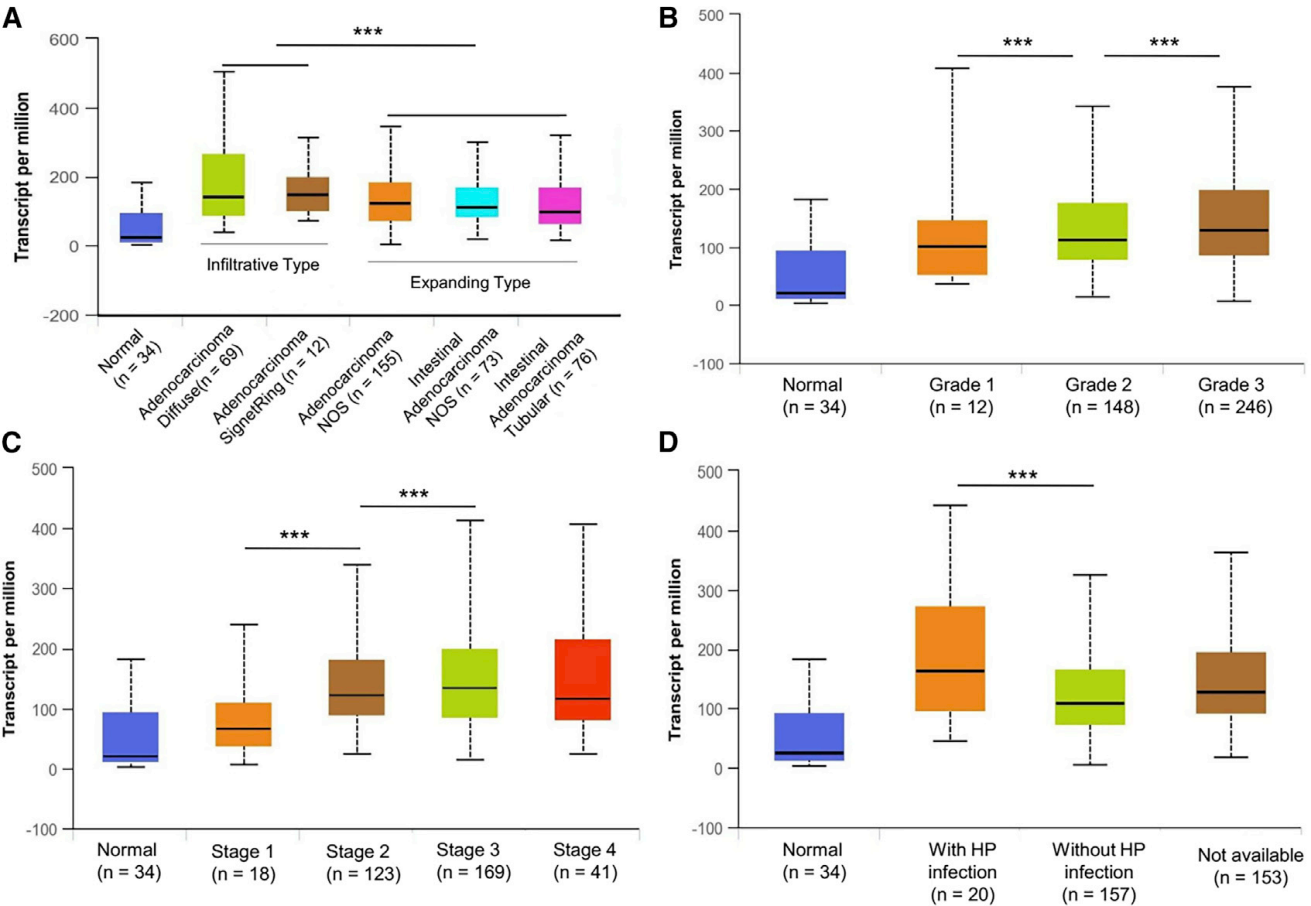
TCGA Data Analysis Revealed That the COL4A1 Expression Was Closely Related to the Clinicopathological Characteristics in GC (A) The COL4A1 expression level in pathological subtypes of infiltrative growth was significantly higher than that in subtypes of expanding growth. (B) The expression level of the COL4A1 gene was positively correlated with malignant grade in GC tissues. (C) The COL4A1 expression was positively correlated with the staging of GC in the tissues. (D) COL4A1 levels in GC patients infected with HP were significantly higher than those in uninfected and undetermined patients. ∗∗∗p < 0.001.

Based on the differential levels of gene expression and extracellular protein secretion of COL4A1 in GC cells from different Ming’s classifications, this study continued to use the data mining tool UALCAN to analyze the association between COL4A1 gene expression and clinicopathological characteristics in GC. The results showed that the COL4A1 expression was significantly higher in the pathological subtypes characterized by infiltrative-type of GC than that characterized by expanding-type GC (Figure 8A). In terms of disease progression, the level of COL4A1 in GC tissues was positively correlated with the malignant grade and staging of GC subjects (Figures 8B and 8C). Moreover, the COL4A1 expression in patients infected with HP is significantly higher than that in uninfected and undetermined GC subjects (Figure 8D). Because HP is an environmental pathogen of GC and the malignant grade and staging of GC are the main pathological indicators of disease progression, it was suggested that the COL4A1 gene may play a role in promoting disease occurrence, accelerating pathological progress in the development of GC.

### Downregulation of COL4A1 Expression Significantly Inhibited the Migration and Invasion of GC Cells

**Figure 9:**
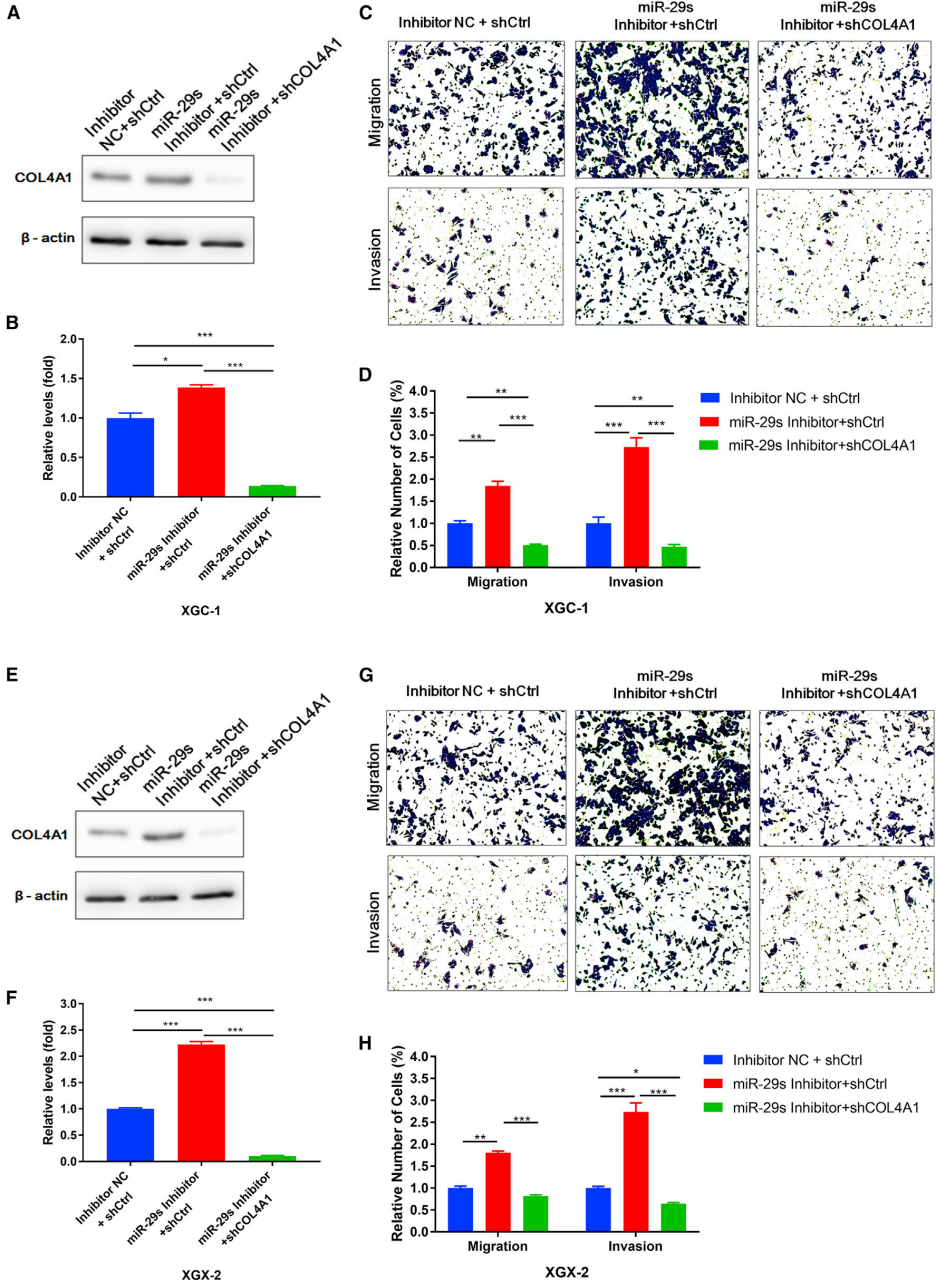
Downregulation of COL4A1 Expression Significantly Inhibits Migration and Invasion of GC Cells ∗∗∗p < 0.001.

In the progress of GC, the migration and invasion of cancer cells are the main causes of disease progression. The results of microarray screening, bioinformatics, cell molecular biology, and clinicopathological correlation analysis suggested that the expression of COL4A1 may influence the migration and invasion of GC cells. In this study, knockdown vectors targeting COL4A1 expression were constructed to induce functional deletion of the COL4A1 gene in GC cells, and then the effects on migration and invasion of GC cells were analyzed by a Transwell experiment. In addition, the COL4A1 expression can be elevated by transfecting the inhibitors of miR-29s. The miR-29s inhibitors and the knockdown vectors of COL4A1 were co-transfected into GC cells to evaluate the biological role of the COL4A1 gene on the whole regulatory network of miR-29s, especially their effect on the migration and invasion of GC cells. The result showed that inhibiting the function of miR-29s could significantly enhance the ability of migration and invasion in GC cells while the COL4A1 protein level was increased. However, the above phenomena caused by the function loss of miR-29s were eliminated when the miR-29s inhibitors and the COL4A1 knockdown vector were co-transfected into GC cells (Figure 9). The results were validated in two GC cell lines from Ming’s classifications. It was indicated that the abnormal higher expression of COL4A1 may be essential to enhance the migration and invasion of GC cells among the target genes of the miR-29s regulatory network.

### Survival Analysis Revealed That High COL4A1 Expression Predicted the Poor Prognosis in GC

**Figure 10:**
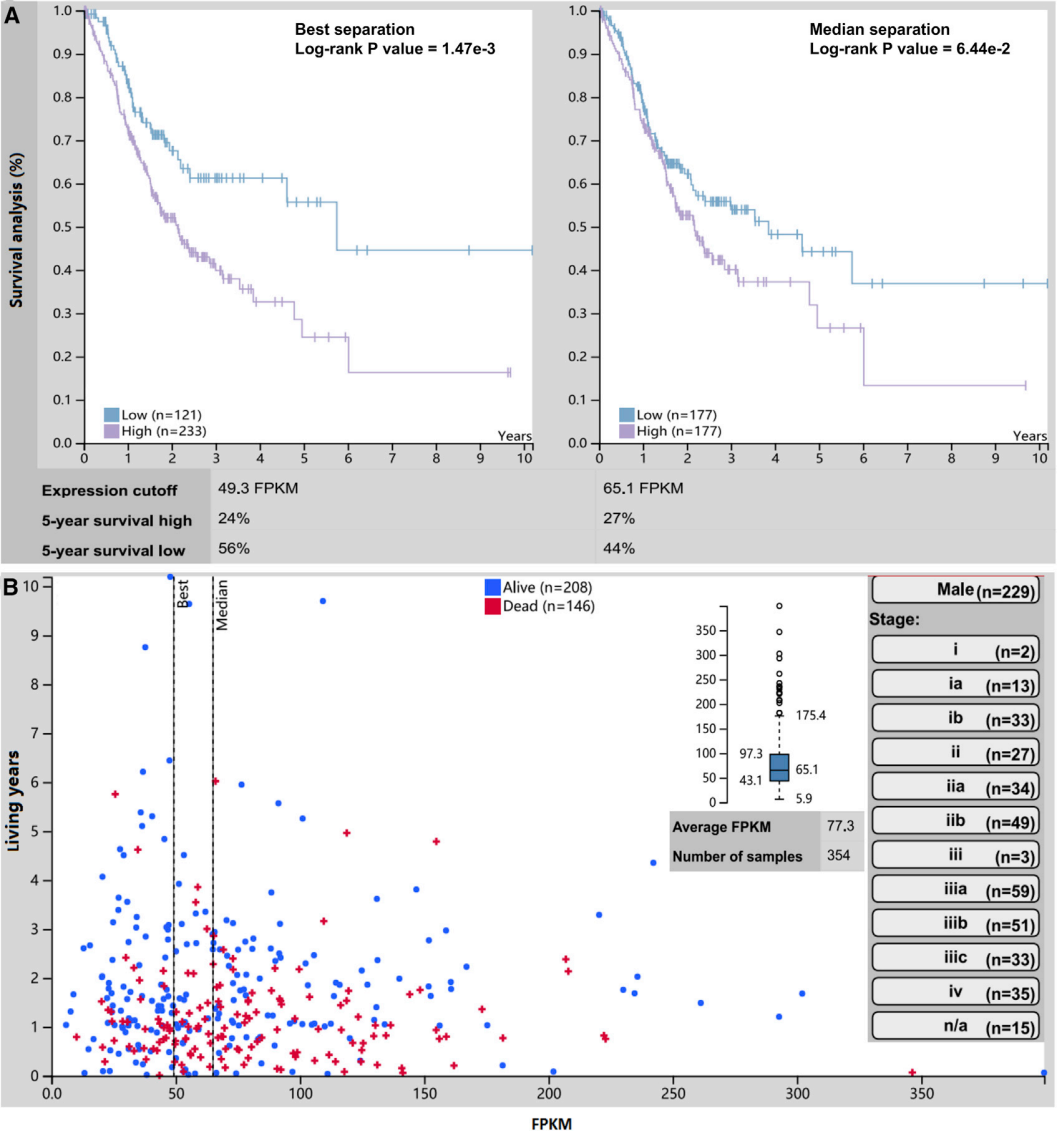
Survival Time Was Significantly Correlated with COL4A1 Expression in GC Patients (A) Survival analysis showed that COL4A1 expression could predict the prognosis of GC patients at certain levels. (B) Information of COL4A1 expression in samples with GC and their clinical features. ∗p < 0.05.

At present, the lack of biological markers that can accurately predict the survival and prognosis of GC patients has been a problem in clinical practices. In this study, COL4A1 gene expression can promote the migration and invasion of GC cells and be correlated with many malignant pathological factors of GC (Figure 8). Therefore, it is necessary to explore the clinical value of COL4A1 gene expression in the prognosis of GC. FPKM (fragments per kilobase of exon per million reads mapped) is a commonly used index for transcript quantification. In this study, the Human Protein Atlas (HPA) online tool was used for TCGA data mining, and FPKM was used as a gene expression index in the 5-year survival analysis of 354 patients with GC. The results showed that the expression level of COL4A1 could reflect the prognosis of GC patients to some extent (Figure 10: best separation, log-rank p = 1.47e−3, cutoff = 49.3 FPKM; median separation, log-rank p = 6.44e−2, cutoff = 65.1 FPKM), suggesting that patients with high expression of COL4A1 had worse prognosis than did those with low COL4A1 expression. The expression level of COL4A1 gene in GC tissue may be a potential biological marker that predicted the prognosis of GC.

## Discussion

GC tissues contain many kinds of cells, such as tumor cells and stromal cells.^18^ GCs have strong heterogeneity, and their histological classification is mainly based on the morphological manifestations of tumor tissues under the microscope.^18^ According to different pathological characteristics, there are many classification criteria at present.^19^^,^^20^ The emphasis of Lauren’s classification has been on the purely structural patterns rather than the behavioral pattern of GC, and Lauren’s classification was given at a time when detection of early GC was relatively difficult.^5^ In Ming’s classification, GC is classified concisely from the biological characteristics such as infiltration and growth pattern, which are the most distinct and invasive to the body.^5^^,^^6^ The prognosis in the two types of GC is also significantly different, which indicates that the classification that combined the biological characteristics of GC has a certain reference value for the evaluation of disease progression. In previous studies, we found that there were significant differences in the expression profiles of miRNAs between expanding-type GC and infiltrative-type GC.^11^. This distinctly differential miRNA expression profile demonstrates that these two types of GC have distinct regulatory mechanisms of gene expression. In this study, the expression of miR-29b/c is low in infiltrative-type GC, but it shows no significantly abnormal expression in expanding-type GC. These two miRNAs have the same seed sequence and belong to the same miRNA family, and other studies have reported that miR-29a also participates in the occurrence of GC. Therefore, this study attempted to explore the regulatory pathway of all members of miR-29s (miR-29a, miR-29b, and mir29-c), which may be involved in the process of different growth patterns of GC.

First, we validated the expression of miR-29s in a large sample of GC patients. The results showed that the expression level of miR-29s in GC tissues was significantly lower than that in corresponding normal gastric epithelium tissues (Figure 1). Then, through cluster analysis of a series of bioinformatics databases, we found that the ECM-receptor interaction pathway is most closely related to the role of miR-29s (Figure 1C). The interaction network between miR-29s and target genes in this pathway is established (Figure 2A). GO (Gene Ontology) enrichment analysis reveals that the functions of target genes are mainly contained in cell adhesion, endothelial development, and microangiogenesis (Tables 1 and 2). It is suggested that ECM organization, cell motility, and cell adhesion are the main biological functions of the target genes of the miR-29s in this pathway (Figures 2B–2D). Oncomine data suggested that COL4A1 may be the key target gene of miR-29s in the ECM-receptor interaction pathway (Figure 3). The molecular mechanisms revealed that miR-29s inhibited gene expression by binding to the 3′ UTR region of COL4A1 gene in GC cells (Figures 4 and 5), and the COL4A1 expression was negatively correlated with the levels of miR-29s in clinical tissues of GC, which strongly indicated that the regulatory network of miR-29b/c-COL4A1 may be involved in the different growth pattern of GC. Downregulation of COL4A1 gene expression can lead to a significant reduction of the S-phase cell ratio in cancer cell cycle and arrested in the G_0_/G_1_ phase.^21^^,^^22^ A recent study has proved that mutation located at the COL4A1 3′ UTR of the miR-29s binding site could cause human cerebral small vessel disease.^23^ COL4A1 protein is the main component of the tissue basement membrane, where many protein molecules formed a complex interaction network, affecting cell movement, cell survival, microangiogenesis, and cell differentiation.^21^ The deposition of COL4A1 protein can activate the ITGB1 signaling pathway in tumor tissues, and then the elasticity and adhesion ability of cancer cells are reduced.^22^ It is easier for cancer cells to disperse and metastasize to adjacent tissues. In this study, we found that the expression level of COL4A1 gene increased gradually with the aggravation of malignancy and the progress of clinical stage in GC (Figure 8). The production of COL4A1 in most GC cell lines is higher than that in gastric epithelial cells (Figures S1A and S1B). Infiltrative-type GC cells secreted higher COL4A1 protein level than did the expanding-type of GC cells (Figure 7), suggesting that the different levels of COL4A1 protein content in extracellular matrix of GC tissue may be one of the reasons for different growth patterns of GC.

It has been reported that HP can secrete invasive protease to degrade and cut E-cadherin and other cell adhesion factors in the basolateral membrane of normal gastric epithelium, thus destroying the protective layer of normal gastric epithelium.^24^, ^25^, ^26^ This process can induce abnormal expression of some autophagy-related miRNAs, triggering regulatory disorders between miRNAs and target genes.^27^ Although the mechanism between HP infection and COL4A1 abnormal expression in GC is not clear, it still indicated that COL4A1 might not only affect the malignancy of GC, but it also plays a biological role in promoting the progression of GC. *In vitro* experiments showed that downregulation of COL4A1 expression significantly inhibited the migration and invasion of GC cells from different growth patterns (Figure 9). Survival analysis showed that the GC patients with high COL4A1 expression had worse prognosis (Figure 10). Combined with the above results, it suggested that the target gene of miR-29s may affect the biological functions of GC cells such as cell motility and cell adhesion through regulating the ECM-receptor interaction pathway, thus promoting the different growth pattern development in GC, suggesting that the target gene of miR-29s, especially the COL4A1 gene, may play an important biological role on GC growth and invasion.

At present, several studies have suggested that miR-29s may be involved in apoptosis, metastasis, microangiogenesis, chemotherapy resistance, and other biological processes of GC (Table S1).^16^^,^^17^ However, the regulatory network of miR-29s is very complex, and the biological regulatory network in GC tissue is a dynamic process. The three members of this family perform not exactly the same functions in cells due to the difference of 3′ terminal sequences. So, which biological pathway is the main way of the miR-29s to function in the development of GC? The above studies did not provide relevant information.

Interestingly, miR-29b/c was significantly lower in infiltrative-type GC than in expanding-type GC, which strongly suggested that the miR-29s might selectively affect some specific biological characteristics during the occurrence and development of GC. It is helpful to elucidate the mechanism that resulted in different growth patterns of GC to explore the relationship between miR-29s expression and Ming’s classification, and to find out more relevant molecules involved in GC cell migration, invasion, and disease prognosis. This is the reason to choose them as the object for the follow-up function analysis. This study found that the ECM-receptor interaction pathway is most closely related to the function of miR-29s (Table 1; Figure 1C). It has been reported that miR-29c can regulate the expression of the ITGB1 gene in the ECM-receptor interaction pathway, and ITGB1 can regulate the degree of the adhesiveness between cells and ECM, thus affecting the metastasis of GC.^28^ This report further proved that the bioinformatics analysis strategy adopted in this study has an important guiding value in clarifying the main pathway of miR-29s in the regulatory network of GC different growth pattern. We further hypothesize that the common target gene of miR-29s may be the main way to play biological roles in GC. Another highlight of this study is to screen the common target gene in both the ECM-receptor interaction and the susceptible gene of the specific GC phenotype. As diffuse GC is classified as infiltrative-type GC in Ming’s classification, this strategy ensures that the subsequent candidate genes (COL4A1, COL3A1, and COL1A2) and Ming’s classification-related miR-29b/c are all associated with the same GC phenotype, which has the same morphological performance in clinical pathology. This strategy is helpful to find the disease biomarkers with clinical value in the malignant degree, clinical progress, and survival prognosis of GC.

Remarkably, the COL4A1 expression was also significantly correlated with the HP infection status in GC patients (Figure 8). Since HP infection often occurs in the early stage of GC, it is reasonable to assume that the abnormal expression of COL4A1 may not only affect the migration and invasion ability of GC cells, but it may also be involved in the induction of HP-related inflammatory stimuli and gastric epithelial carcinogenesis, which may lead to different histological origins of GC in early stage of different growth patterns. Moreover, it is reported that COL4A1 protein is mainly distributed in the stromal region around the cancer cell site, which can degrade E-cadherin and other adhesion molecules through the AKT signaling pathway to trigger tumor sprouting and promote tumor invasion.^29^ This suggested that upregulation of the COL4A1 may had an important influence on cell behavior in the tumor microenvironment. The effect of miR-29s and COL4A1 gene expression on the regulation of the growth pattern of GC in the process of HP infection is an interesting follow-up research field. The hypothesis proposed by the results of this study helps to further understand and explain the molecular mechanism of the origin of different growth patterns of GC.

### Conclusion

In summary, miR-29s, which are related to different growth patterns of GC, affected the biological functions of GC cell migration and infiltration by targeting the COL4A1 gene in the pathway of ECM-receptor interaction, and participate in the formation of GC different growth patterns. The COL4A1 is expected to be a biological marker with the potential clinical value of predicting malignant grade, progression stage, and disease prognosis of GC.

## Materials and Methods

### Clinical Specimens

A total of 93 GC subjects receiving operation in Zhongshan Hospital were included in this study. The normal gastric tissues were 5.0 cm from the matched tumor boundary and used as controls. After routine hematoxylin and eosin (H&E) staining, the histopathological diagnosis and characteristics of tumors in tissues were evaluated by two experienced pathologists. Tumors were staged according to the TNM staging system (7th ed.). Histological grade was based on the National Comprehensive Cancer Network (NCCN) Clinical Practice Guideline of Oncology (v.1.2012). All of the specimens were rapidly frozen in liquid nitrogen after being collected from the body and then stored at −80°C. None of the included patients received preoperative radiotherapy, chemotherapy, or other antineoplastic treatments before the specimens were obtained. All aspects of this study were approved by the Medical Ethics Committee of Zhongshan Hospital, and written informed consents were obtained from all subjects.

### Cell Culture

The human gastric epithelial cell line (GES-1) was obtained from the Cancer Institute and Hospital of the Chinese Academy of Medical Sciences (Beijing, China). The human GC cell lines (MKN-45, AGS, HGC-27, MGC80-3, MKN-28, BGC-823, and SGC-7901) and the human embryonic kidney 293 cell line (HEK293T) were purchased from the Shanghai Institute of Biochemistry and Cell Biology, Chinese Academy of Sciences (Shanghai, China). The new GC cell lines of Ming’s classification were established and characterized in our laboratory as previously reported.^11^^,^^30^ The XGC-1 cell line originated from infiltrative-type GC and the XGC-2 cell line was established from expanding-type GC.^11^ In this study, the GES-1 cell line was used as a control. All of the cells were cultured in fluid medium (HyClone, Logan, UT, USA) with 10% fetal bovine serum (FBS) and incubated at 37°C in a humidified atmosphere with 5% CO_2_.

### RNA Extraction and Quantitative Real-Time PCR

The isolation of the total RNA from both cell lines and frozen tissues were used with TRIzol reagent (Ambion, Carlsbad, CA, USA) according to the manufacturer’s instructions. RNA quantity and purity were evaluated by a Multiskan GO 1510 spectrophotometer (Thermo Fisher Scientific, Vantaa, Finland). All quantitative real-time reverse transcription polymerase chain reaction (RT-PCR) process was performed in a CFX96 real-time PCR system (Bio-Rad, Hercules, CA, USA). First, a miDETECT A Track miRNA qRT-PCR starter kit (Ribobio, Guangzhou, China) was used for synthesizing cDNA of small RNAs and evaluating miRNAs expression as previously reported.^11^^,^^12^ Second, the synthesizing cDNA of mRNA was used with the ReverTra Ace qPCR RT master mix (Toyobo, Osaka, Japan) from 1 μg of total RNA, and the levels of mRNA expression were detected using PowerUp SYBR Green master mix (ABI/Life Technologies, Austin, TX, USA) according to the manufacturer’s protocol. The specific primers of miR-29s were synthesized by Ribobio (Guangzhou, China). The investigators were blinded to the results of clinical and pathological diagnoses. All targets and references gene were amplified in triplicate wells. The 2^−ΔΔCt^ method was used to calculate the relative levels of targets.

### Bioinformatics Analysis of the miRNA Pathway and Risk Genes in Diffuse GC

GO enrichment analysis and pathway function analysis of the miR-29s biological pathway were carried out in combination with multiple miRNA bioinformatics databases (starBase, miRNA Base, TarBase, TargetScan, and DIANA Tools) to identify the biological role of differentially expressed miR-29s in GC cell lines from Ming’s classification. The analysis flow was carried out according to the protocol of previous reports.^11^^,^^31^, ^32^, ^33^ Due to the previous miRNA microarray results and differential expression of miR-29b/c in Ming’s cell lines,^11^ it is important to find the targets of miR-29s associated with infiltrative-type GC to elucidate their biological mechanism in GC development. In Ming’s classification, diffuse GC is classified as infiltrative-type GC.^5^ Therefore, we identified the higher expression gene that mostly related to diffuse GC by using the Oncomine database and then screened the target genes in the most significant biological pathway related to miR-29s by Venn diagram analysis. Thus, the target of miR-29s that most related to Ming’s classification may be found in diffuse GC.

### Prediction of Target Gene Binding Sites and the LUC Reporter Gene Assay

The 3′ UTR binding sites of target genes were predicted using the online database miRDB (http://mirdb.org/miRDB/). Then, the 3′ UTR DNA fragments of WT and MUT were constructed according to the predicted binding sites. The fragments were introduced into the front of the LUC gene expression sequence to construct the recombinant plasmid as previously reported.^12^ The miRNA-specific expression reporter vector system (pMIR-REPORT miRNA expression reporter vector system, Applied Biosystems) is used in this part of the experiment. The system consists of a LUC reporter vector and another matching reference plasmid for β-galactosidase (β-gal) reporting. For LUC reporter assays, 8 μg of LUC reporter plasmid, 8 μg of β-gal expression vector, and 10 μL of 20 μM mimics, inhibitors, or scrambled negative control (NC) RNA were transfected into HEK293T cells in six-well culture plates, respectively. Here, the β-gal vector was used as a transfection control. At 36 h after transfection, cells were collected and assayed using a TriStar2 LB 942 multimode microplate reader system (Berthold Technologies, USA).

### Synthesis and Transfection of miR-29s-Specific Inhibitors or Mimics, and Western Blotting Analysis

The sequence information of miR-29s was obtained from an international general information database of miRNAs (miRBase, http://www.mirbase.org). The specific inhibitors and mimics of miR-29s were synthesized and purified by GenePharma (Shanghai, China). In brief, cells were seeded into a six-well plate 24 h prior to transfection. Transfection with miR-29s mimics or inhibitors was performed using Lipofectamine 3000 (Invitrogen, USA) according to the manufacturer’s protocols. For each well, equal doses (10 μL of 20 μM) of miR-29s mimics, inhibitors, or NC RNA (GenePharma, Shanghai, China) were used. The cells were harvested at 24 h after transfection for quantitative real-time RT-PCR analysis and 48 h for WB, respectively. The relative expression of COL4A1 protein was assessed by WB analysis, and the expressions were normalized to β-actin. Each sample was separated on a 10% SDS-PAGE gel and then transferred onto polyvinylidene fluoride (PVDF) membranes. The membranes were blocked with PBS containing 5% fat-free dried milk at room temperature for 1 h and incubated at 4°C overnight with anti-COL4A1 (1:1,000; Abcam, UK) and anti-β-actin (1:2,000; Sigma, USA) antibodies. After being incubated with the appropriate horseradish peroxidase (HRP)-conjugated secondary antibodies (Thermo Fisher Scientific), the bands were developed using an enhanced chemiluminescence (ECL) system (Amersham Biosciences, Buckinghamshire, UK).

### Identification of COL4A1 Expression in GC and Its Correlation with miR-29s

To further explore the expression of human COL4A1 in GC, a data mining tool, UALCAN (http://ualcan.path.uab.edu/index.html), was used to identify COL4A1 expression in GC by mining TCGA database.^34^ A comparative analysis of large samples was conducted to further verify the biological role of this gene in the pathogenesis of GC. In addition, in order to verify whether the interaction between miR-29s and COL4A1 is involved in the pathogenesis of GC, the correlation between miR-29s level and COL4A1 expression was analyzed in 93 GC subjects receiving operations in Zhongshan Hospital, so as to clarify whether the miR-29s and COL4A synergistically participate in the occurrence and development of GC.

### Meta-Analysis of COL4A1 Expression and COL4A1 Secretion Detection in GC

In order to eliminate the random errors of independent studies, the Oncomine database was used to perform a meta-analysis of COL4A1 expression in all current microarray studies of GC. The expression of COL4A1 in GC cell lines of Ming’s classification were detected by both quantitative real-time RT-PCR and WB techniques, which is helpful to further study the role of COL4A1 in the development of GC, especially in the molecular mechanism of GC differential growth modes. In addition, the secretion of COL4A1 in supernatant of cell culture medium was determined using an ELISA kit (Cusabio, Wuhan) according to the manufacturer’s protocols. Sample was added into appropriate wells and agitated gently at room temperature. The optical density (OD) value at 450-nm wavelength was measured to estimate the sample concentration.

### Analysis of COL4A1 Level and Clinicopathological Characteristics in GC Tissue

Identification of the role of COL4A1 in histological classification and GC progression was the foundation for subsequent cytological function experiments, so we used TCGA data mining tool UALCAN to further analyze the correlation between COL4A1 expression level and clinicopathological characteristics, such as histological classification, tumor progression stage, and HP infection statues in GC.

### Cell Transfection and Transwell Assay

The production of lentivirus carrying short hairpin RNA (shRNA) was constructed as previously reported^35^ to knock down the expression of COL4A1 in GC cells. For transduction, GC cells were infected for 24 h with 10 μL of virus suspension containing 8 μg/mL Polybrene (Sigma, VT, USA). The cell functions were tested using a Transwell Boyden chamber (Costar, USA) with polycarbonate membranes (8-μm pore size) either coated with 10 μg of Matrigel (BD Biosciences, Bedford, MA, USA) per well (for invasion assays) or left uncoated (for migration assays) in serum-free medium containing 10 g/L bovine serum albumin on the bottom of the upper compartment. The cells were suspended in serum-free DMEM medium at a total amount of 5 × 10^5^ cells; simultaneously, 0.5 mL of DMEM with 10% FBS was added to the lower compartment served as a chemoattractant. Non-invading cells were removed with cotton swabs after 36 h. Invaded cells were fixed with 90% ethanol for 15 min at room temperature and stained with 0.1% crystal violet solution. Images of migrated and invaded cells were captured by a photomicroscope and quantified by blind counting with five fields per chamber.

### Clinical Value Assessment of COL4A1 in GC

The HPA database (https://www.proteinatlas.org/) is a collection of RNA and protein expression profiles in human cancer tissues.^36^^,^^37^ It provides important pathological resources for the study of oncoprotein and the discovery of clinical biomarkers. The distribution, localization, and expression level of COL4A1 protein in GC tissue can be preliminarily determined by searching the immunohistochemical information of the cancer tissue protein. Because the HPA database integrates some information of TCGA database, it can be used for survival analysis between gene expression and clinical prognosis. Using this database as a tool can not only evaluate the role of COL4A1 in pathogenesis and progression, but it also helps to clarify the biological significance and clinical value of this target of miR-29s in GC.

### Statistical Analysis

Statistical Program for Social Sciences (SPSS, USA) 17.0 software is used to analyze all of the data. The different value between the experimental group and the control group was analyzed by Student’s t test; the correlation between the expression level of miR-29s and COL4A1 was analyzed by Spearman correlation analysis; the survival analysis was conducted by Kaplan-Meier method and a log rank test; the difference was considered to be statistically significant when p < 0.05 in this study.

## Author Contributions

J.Cheng contributed to study design, analysis of results, and writing the manuscript. J.Cheng, H.Z., L.W., W.Z., and X.C. conducted the experiments. J.H. and J.Z. contributed to the analysis of results. J.Cai designed this study and reviewed the manuscript. All authors read and approved the final manuscript.

## Conflicts of Interest

The authors declare no competing interests.

## Supplemental Information

Document S1. Figure S1 and Tables S1 and S2

Document S2. Article plus Supplemental Information
