# Supplementary material for: Identification of the Combinatorial Effect of miRNA Family Regulatory Network in Different Growth Patterns of GC
Source: Mol Ther Oncolytics. 2020 Mar 30;17:531–46. doi: 10.1016/j.omto.2020.03.012 (PMC7321821; doi:10.1016/j.omto.2020.03.012)
Supplement: Document S1. Figure S1 and Tables S1 and S2 [file mmc1.pdf]

OMTO, Volume *17*

Supplemental Information

Identification of the Combinatorial Effect of miRNA Family Regulatory Network in Different Growth Patterns of GC

Jia Cheng, Huiqin Zhuo, Lin Wang, Wei Zheng, Xin Chen, Jingjing Hou, Jiabao Zhao, and Jianchun Cai

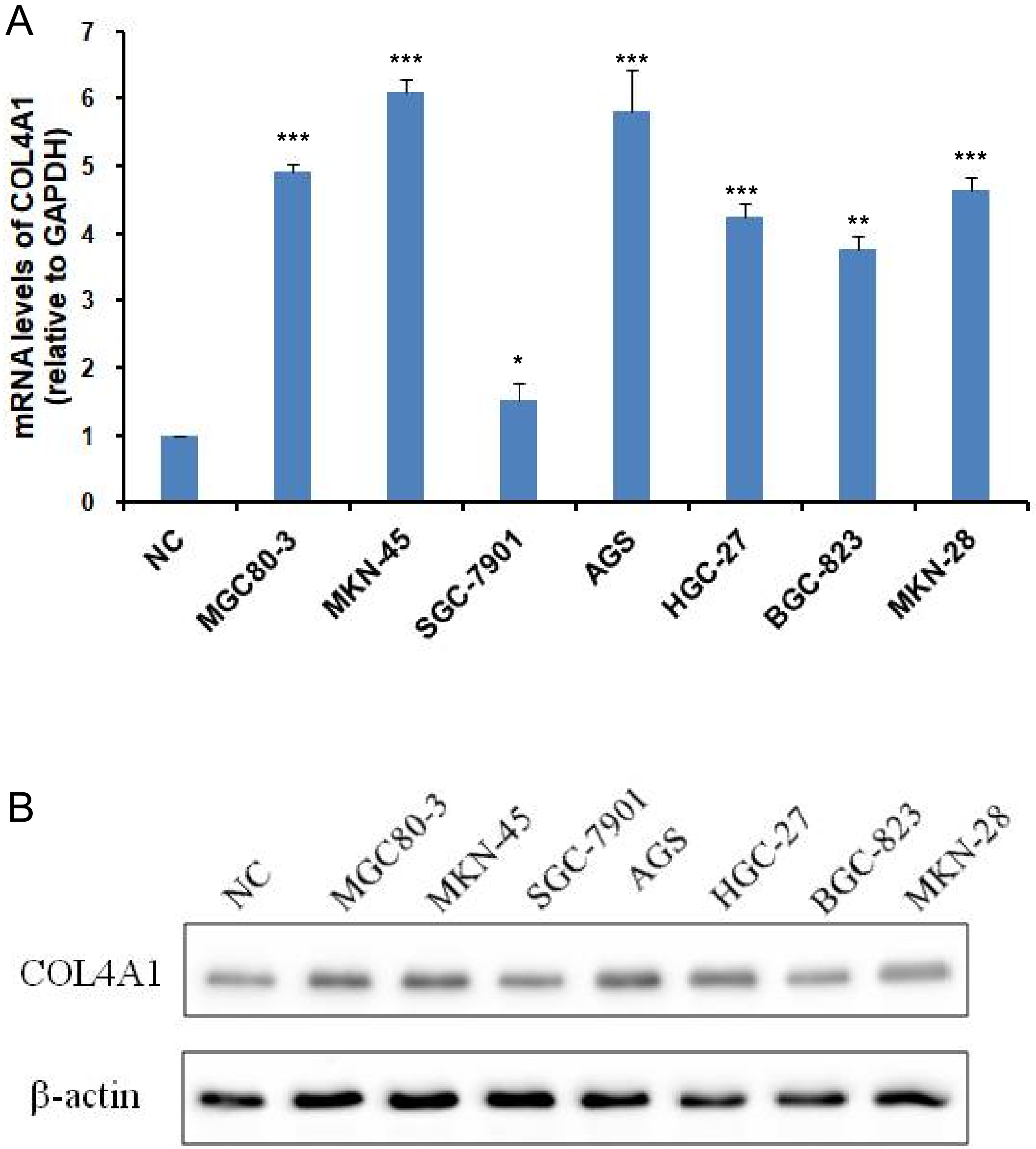

Supplementary Figure 1. The expression status of COL4A1 in cell lines. GES-1 was used as NC. (A) The mRNA levels of COL4A1 in cell lines (B) Protein levels of COL4A1 in cell lines. **P* < 0.05, ***P* < 0.01, ****P* < 0.001.

Supplementary Table 1 A review of the relationship between gastric cancer and miR-29s

Study	Key findings
Wei et al, 2018	Expression of miR-29 was closely related to TNM stage, differentiation degree of cancer cells
Li et al, 2017	Methylation-induced downregulation and gastric tumor suppressive role of microRNA-29b through targeting LASP1
Yu et al, 2017	MicroRNA-29c inhibits cell proliferation by targeting NASP in human GC
Zhang et al, 2016	Cell-derived microvesicles mediate the delivery of miR-29a/c to suppress angiogenesis in gastric carcinoma
Kong et al, 2016	Up-regulation of miR-29b suppresses the proliferation and migration of GC cells by targeting KDM2A
Vidal et al, 2016	DNMT3A is controled by miR-29c expression in GC.
Zhang et al, 2015	MicroRNA-29s could target AKT2 to inhibit GC cells invasion ability
Zhao et al, 2015	Enforced miR-29a expression inhibited cell proliferation and suppressed tumor metastasis in GC
Cui et al, 2015	Deregulation between mir-29b/c and DNMT3a is associated with silencing of the CDH1 in GC
Liu et al, 2015	MiR-29a inhibits migration and invasion in part via direct inhibition of Robo1 in GC cells
Han et al, 2015	MicroRNA-29c-mediates initiation of gastric carcinogenesis by directly targeting ITGB1
Wang et al, 2015	Chemotherapy-induced miR-29c/catenin-δ signaling suppresses metastasis in GC
Wang et al, 2015	Hsa-miR-29 level was associated with the higher overall survival rate
Chen et al, 2014	MiR-29a suppresses growth and invasion of GC cells by targeting VEGF-A
Chen et al, 2014	Mir-29b reduces cisplatin resistance of GC cell by targeting PI3k/Akt pathway
Gong et al, 2014	MiR-29 family might be employed as novel prognostic markers and therapeutic targets of GC
Ma et al, 2013	MiR-29c is involved in targeted regulation of Mcl-1 and related to biological behavior of GC
Matsuo et al, 2013	Decreased expression of miR-29c may confer a growth advantage on tumor cells via aberrant expression of RCC2
Saito et al, 2013	Downregulated microRNA-29c is restored by celecoxib in human GC cells

Cui et al, 2011	Mir-29a inhibits cell proliferation and induces cell cycle arrest through the downregulation of p42.3
Lang et al, 2010	MicroRNA-29s inhibit cell proliferation, migration, and invasion of GC cells by targeting CDC42

Supplementary Table 2 The sequence of primers and siRNA

**Name**	**Sequence**
miR-29a inhibitor	5'-UAACCGAUUUCAGAUGGUGCU-3'
miR-29b inhibitor	5'-AACACUGAUUUCAAAUGGUGCUA-3'
miR-29c inhibitor	5'-UAACCGAUUUCAAAUGGUGCUA-3'
miRNA inhibitor NC	5'-CAGUACUUUUGUGUAGUACAA-3'
miR-29a mimic（double strands）	5'-UAGCACCAUCUGAAAUCGGUUA-3'
	3'- UUAUCGUGGUAGACUUUAGCCA-5'
miR-29b mimic（double strands）	5'-UAGCACCAUUUGAAAUCAGUGUU-3'
	3'- UUAUCGUGGUAAACUUUAGUCAC-5'
miR-29c mimic（double strands）	5'-UAGCACCAUUUGAAAUCGGUUA-3'
	3'- UUAUCGUGGUAAACUUUAGCCA-5'
miRNA mimic NC	5'-CAGUACUUUUGUGUAGUACAA-3'
GAPDH primer-F	5'-AGAAGGCTGGGGCTCATTTG-3'
GAPDH primer-R	5'-AGGGGCCATCCACAGTCTTC-3'
COL4A1 primer-F	5'-CCAGGGGTCGGAGAGAAAG-3'
COL4A1 primer-R	5'-GGTCCTGTGCCTATAACAATTCC-3'
